# Important Clinical Features of Japanese Spotted Fever

**DOI:** 10.4269/ajtmh.17-0576

**Published:** 2018-07-02

**Authors:** Masamitsu Noguchi, Shizuka Oshita, Naohisa Yamazoe, Mitsukazu Miyazaki, Yousuke C. Takemura

**Affiliations:** 1 Department of Internal Medicine, Minami-Ise Municipal Hospital, Mie, Japan;; 2 Department of Family Medicine, Mie University Graduate School of Medicine, Mie, Japan;; 3 Department of Medical Technology, Minami-Ise Municipal Hospital, Mie, Japan;; 4 Department of Family Medicine, Mie University School of Medicine and Graduate School of Medicine, Mie, Japan

## Abstract

Japanese spotted fever (JSF) is a zoonosis transmitted by ticks carrying the pathogen *Rickettsia japonica*. The classic triad of JSF symptoms is high fever, erythema, and tick bite eschar. About 200 people in Japan develop the disease every year. Japanese spotted fever is also a potentially fatal disease. At Minami-Ise Municipal Hospital in Japan, 55 patients were diagnosed with JSF from 2007 to 2015, which was equivalent to 4.3% of the total JSF cases in Japan. In this retrospective study, we examined the medical records of these 55 JSF cases. Fever, erythema, eschar, and elevated C-reactive protein (CRP) are characteristic clinical features of the disease. We confirmed four of these in the reviewed cases; however, eschar was not present in occasional cases. We confirmed that eosinopenia appeared in nearly all cases. Using fever, erythema, elevated CRP, and eosinopenia in diagnostic screening, our positivity rate was 90.9%. In our clinical practice, including eosinopenia improves the initial diagnosis of JSF.

## INTRODUCTION

Japanese spotted fever (JSF) was first described by Mahara et al.^1^ in 1984. Japanese spotted fever emerges mainly during warm seasons, from April to November.^2,3^ The disease occurs mainly in warm areas along the Pacific side of southwest and central Japan^2,4–6^; there have also been reports of cases along the Sea of Japan coast and Korea.^7–9^ In Mie Prefecture, JSF has been reported on the south side of the Miya River,^10^ an area that includes the town of Minami-Ise. Over a nearly 8-year period from May 2007 to January 2015, there were 1,276 cases of JSF reported in Japan, with 277 cases in Mie Prefecture (21.7% of the total for Japan) and 55 cases at Minami-Ise Municipal Hospital (4.3% of the total for Japan).

Japanese spotted fever is caused by *Rickettsia japonica*. The disease develops in humans approximately 2–8 days after being bitten by a tick carrying the pathogen.^2^ The main characteristic clinical features of JSF are high fever, erythema with no pain or itching, and tick bite eschar.^1,2^ Erythema also appears on the extremities and trunk, as well as on the palms and soles of the feet.^2^ The main laboratory findings among JSF cases include leukocytosis or leukopenia, thrombocytopenia, elevated C-reactive protein (CRP), and elevated liver enzymes.^2,3^

Treatment is generally with tetracycline, which is remarkably effective for JSF.^3^ Treatment with steroids is also effective.^11^ In previous reports, complications include disseminated intravascular coagulation (DIC), multiple organ failure, meningoencephalitis, central nervous disorders, and acute respiratory distress syndrome, among others.^3,12^ And, there have been reports of severe or fatal cases.^2,3,13,14^

The classic triad of JSF symptoms is high fever, erythema, and tick bite eschar. The purpose of our study is to elucidate new clinical findings, which are useful for early diagnosis and treatment of JSF.

## MATERIALS AND METHODS

We included 55 patients diagnosed with JSF at Minami-Ise Municipal Hospital from May 2007 to January 2015. All patients were residents of Minami-Ise. We therefore investigated symptoms, signs, and laboratory results from the time of first contact to the end of treatment among JSF cases, by a review of medical records retrospectively.

The definitive diagnosis of JSF is based on laboratory data alone; detection of *R. japonica* antibody titers and/or *R. japonica* DNA from patient blood and/or eschar samples is by polymerase chain reaction (PCR). Measurement of antibody titers is carried out manually by serologic testing, with indirect immunofluorescence assay using *R. japonica* (YH strain). The PCR method is based on the Prevention of Infectious Diseases and Medical Care for Infectious Patients Act of the Ministry of Health, Labor and Welfare of Japan. Conventional PCR is performed using primers targeting *R. japonica* DNA on a G-storm GS4 (Somerton Biotechnology Center, Somerset, United Kingdom); there are two types of primers. One contains primers R1 (5′-TCAATTCACAACTTGCCATT-3′) and R2 (5′-TTTACAAAATTCTAAAAACC-3′) which detect spotted fever group *Rickettsia* and typhus group *Rickettsia*. The other contains primers Rj5 (5′-CGCCATTCTACGTTACTACC-3′) and Rj10 (5′-ATTCTAAAAACCATATACTG-3′) which specifically amplify only *R. japonica*. Measurement of antibody titers and PCR method are not carried out at our hospital; therefore, we send all patient samples for testing to an external laboratory, the Mie Prefecture Health and Environment Research Institute.

This study was approved by the Research Ethical Committee of Mie University Graduate School of Medicine (No. 1476).

## RESULTS

Patient characteristics are shown in Table 1. The median patient age was 77 (24–92) years. A total 81.8% of patients were hospitalized and managed. And 80.0% had their first contact with our hospital within 5 days from symptom onset. Signs and symptoms are listed in Table 2. From the onset to the first contact, 52 patients had a symptom of fever. The average value of fever at the first contact was 38.3°C for males and 38.0°C for females. And, only one patient did not have fever or erythema. Among the 45 patients who were hospitalized and treated with antipyretics, 64.4% (29 of them) had fever of a remittent type; nearly all peaks of fever were between the evening and the next morning. The degrees and duration of fever, and the duration of fever after starting antipyretic therapy are presented in Table 3. Three cases had no fever at any time during the clinical course. (38 of 45) 84.4% of patients with fever were started on defervesce within 5 days of antipyretic therapy.

**Table 1:** Patient characteristics

	*N*	%
Incidence by age*		
< 65 years	0.20	–
≥ 65 years	0.91	–
Gender		
Male	27	49.1
Female	28	50.9
Comorbidities		
Hypertension	22	40.0
Dyslipidemia	12	21.8
Diabetes mellitus	3	5.5
Alzheimer-type dementia	1	1.8
Bronchial asthma	1	1.8
Gastric cancer	1	1.8
Hepatocellular carcinoma	1	1.8
Chronic heart failure	1	1.8
Old myocardial infarction	1	1.8
Duration from symptom onset to initial diagnosis (days)†		
1	3	5.5
2	7	12.7
3	15	27.3
4	10	18.2
5	9	16.4
6 or more	5	9.1
Unknown	6	10.9

* Per 1,000 population.

† Average from onset to initial diagnosis was 2.7 days.

**Table 2:** Clinical features at the first contact

	*N*	%
Symptoms		
Fever from the onset to the first contact	52	94.5
Malaise	22	40.0
Gastrointestinal symptoms*	21	38.2
Headache	9	16.4
Arthralgia	7	12.7
Sore throat and cough	6	10.9
Myalgia	3	5.5
Aware of tick bite†	6	10.9
Signs		
SBP ≤ 90 mm of Hg	1‡	2.0
Impaired consciousness§	3‖	5.6
Erythema	51	92.7
Eschar	34	61.8

SBP = systolic blood pressure. Note: patient total = 55.

* Gastrointestinal symptoms include anorexia, abdominal pain, vomiting, and nausea.

† Patients were aware that they had been bitten by a tick.

‡ *N* = 50.

§ One case could not be diagnosed because the patient had dementia.

‖ *N* = 54.

**Table 3:** Degree and duration of fever, and duration required for defervescence

Fever*	*N*	%
From the onset to the first contact		
≥ 39°C	21	38.2
≥ 38°C	50	90.9
≥ 37.5°C	52	94.5
Fever duration from initiation of treatment to defervescence†		
Within 3 days	17	37.8
Within 4 days	32	71.1
Within 5 days	38	84.4

* Total patients = 55.

† Total patients = 45.

Most patients arrived at our hospital in a state of clear consciousness. Three patients had mildly impaired consciousness and one had dementia. Hypotension was observed in one case; however, this patient did not have impaired consciousness or oliguria, and therefore did not meet the criteria for shock.

At the first contact, erythema was observed in most patients. And, in 80.0% (44 of 55) of patients, erythema appeared in the extremities. One patient had no erythema at any time during the clinical course. In another patient, erythema had changed to pigmentation. Eschar was present among most of the patients. The positivity rate for eschar PCR results was 88.2% (30 of 34 patients), whereas at the same time, the positivity rate for blood PCR was 45.3% (24 of 53 patients). Only six patients realized that they had been bitten by a tick; most patients did not notice the bite.

**Figure 1.**
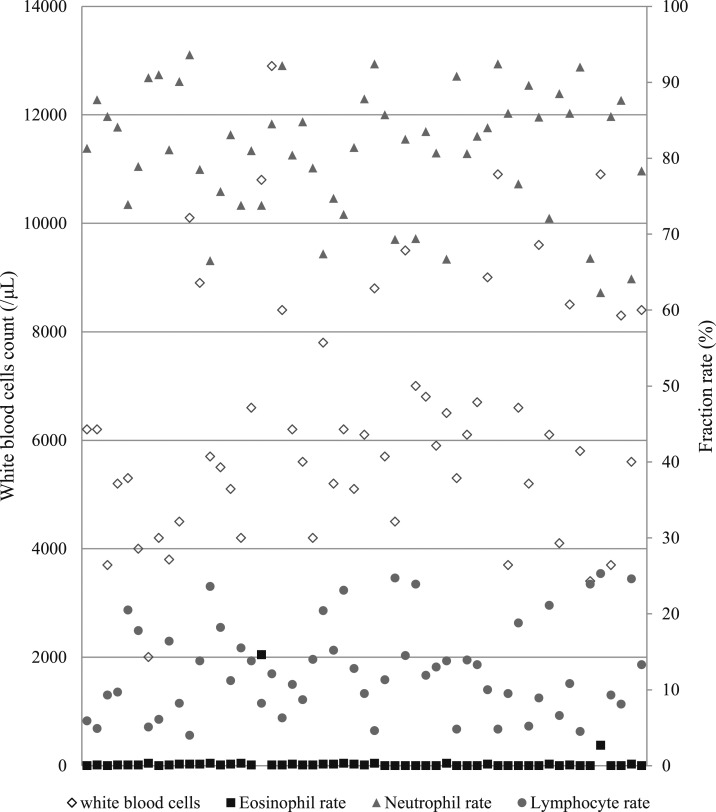
Distribution of white blood cells count and fraction rate.

Laboratory examination results are shown in Table 4. C-reactive protein was at high levels in all cases tested. A total of 81.8% of patients had elevated liver enzymes and 52.7% had thrombocytopenia. All cases had eosinopenia, except for those with an allergic reaction. The eosinophil rate are shown in Figure 1.

**Table 4:** Rapid laboratory test data at the first contact

	Reference interval*	Mean	Range
T-Bil (mg/dL)	0.2–1.3	0.73	0.3–1.8
AST (IU/L)	10–35	60.7	15–226
ALT (IU/L)	10–35	38.3	7–195
LDH (IU/L)	110–225	310.1	170–643
CK (IU/L)	20–200	289.1	46–3,086
BUN (mg/dL)	9.0–22.0	18.1	7.4–52
SCr (mg/dL)	0.50–1.10	0.878	0.41–2.53
eGFR (mL/min)	≥ 60	63.84	20.4–127.8
CRP† (mg/dL)	≤ 0.3	7.745	0.6–21.97
WBC (/μL)	4,000–9,000	6,405	2,000–12,900
Neutrophil count (/μL)	1,800–6,390	5,194.1	1,812–10,900.5
Neutrophil rate (%)	45–71	81.0	62.3–93.6
Lymphocyte count (/μL)	1,000–4,050	803.2	102.0–2,757.7
Lymphocyte rate (%)	25–45	12.7	4.0–25.3
Monocyte count (/μL)	40–450	343.5	54–1,228.5
Monocyte rate (%)	1–5	5.5	1.0–18.9
Eosinophil count (/μL)	40–450	40.00	0–1,576.8
Eosinophil rate (%)	1.0–5.0	0.4	0–14.6
Basophil count (/μL)	0–40	24.6	0–259.2
Basophil rate (%)	0–1	0.4	0–2.4
PLT (/μL)	130,000–140,000	133,000	42,000–239,000

ALT = alanine aminotransferase; AST = aspartate aminotransferase; BUN = blood urea nitrogen; CK = creatine kinase; CRP = C-reactive protein; eGFR = estimated glomerular filtration rate; LDH = lactate dehydrogenase; PLT = platelets; SCr = serum creatinine; T-Bil = total bilirubin; WBC = white blood cells. Note: patient total = 55.

† *N* = 54.

* Reference intervals refer to those of Minami-Ise Municipal Hospital.

In our study, there were two severe cases of JSF. One case was DIC. Another patient had takotsubo cardiomyopathy; this patient died.

## DISCUSSION

Japanese spotted fever was described for the first time in 1984 in Tokushima Prefecture of Japan. As of 2017, JSF had appeared in 28 Japanese prefectures.^15^ In Mie Prefecture, JSF has been reported since 2005. Mie Prefecture is a predilection site for JSF, and the town of Minami-Ise has the highest incidence of disease in the area. The occurrence area of JSF and other tick-borne diseases has been expanding, especially in warm regions of Japan.^1,13,15^ Therefore, we consider that it is important to inform physicians and medical institutions about the disease, even in regions where no JSF has been observed.

The classic triad of JSF symptoms is fever, erythema, and eschar. In our study, almost all patients had fever. As described earlier, remittent fever was common,^2^ and most patients defervesced within 5 days of treatment. Japanese spotted fever and scrub typhus are very similar diseases, as has been previously reported; patients with scrub typhus defervesce within 24 hours of the treatment.^16^ Of the classic triad of symptoms, eschar was found infrequently, in only about 60% of our patients. However, the positivity rate of PCR for definitive diagnosis of JSF was higher for eschar samples than for whole blood.

Regarding laboratory results, CRP was nearly always elevated in our cases. This is considered to reflect the status of the infection. Eosinopenia was observed in all patients except two, who were diagnosed with allergic disorders based on other test results. Eosinopenia is considered a good marker of bacteremia and is suspected of increasing mortality.^17^ Elevated liver enzymes and thrombocytopenia were also observed among our patients, as well as high or low leukocyte blood counts.

Based on these results, we consider fever, erythema, elevated CRP, and eosinopenia to be good markers of JSF, even when there is no eschar present. In our study, the positivity rate of diagnosis based on these four features was 90% or more for each item, and 90.9% of patients had all four features. There were no patients with JSF who did not present with all four symptoms. With respect to these features in a primary care setting, a comprehensive early diagnosis should be made in conjunction with other clinical symptoms and laboratory findings; it is important to initiate early treatment. In the past, Funato et al.^18^ have described about the relationship between JSF and eosinopenia in their English abstract; the text is only in Japanese and is a nonnumerical report about eosinopenia. In our study, we were able to deal with larger numbers of subjects than the previous study and did quantify eosinopenia in English.

To our knowledge, the JSF fatality rate has not yet been described in any peer-reviewed journals published in English. Rocky Mountains spotted fever (RMSF), which occurs in the United States, is a similar disease to JSF. The RMSF fatality rate has been described as less than 1%.^19^ In our study, one patient died. We consider that treatment and management of JSF are possible, even at smaller medical facilities such as our small hospital which was responsible for community medicine, if an initial diagnosis can be accurately made and prompt treatment initiated.

The limitation of this study is that we could not generalize the results to larger or other populations because this research was performed retrospectively at one rural hospital in Japan. No available analysis of specificity is another limitation in this study.

In conclusion, in addition to fever, erythema, and elevated CRP, eosinopenia is an effective and useful tool that can be used for early diagnosis of JSF, even when there is no eschar present.
